# ASO Author Reflections: Single-Dose Neoadjuvant Pembrolizumab in Resectable Metastatic Melanoma

**DOI:** 10.1245/s10434-025-18456-6

**Published:** 2025-09-29

**Authors:** Mohammad Saad Farooq, Neha Shafique, Giorgos C. Karakousis

**Affiliations:** Division of Endocrine and Oncologic Surgery, Hospital of the University of Pennsylvania, Philadelphia, PA USA

## Past

Recent clinical trials have reported superior outcomes using neoadjuvant immunotherapy compared with surgery followed by adjuvant immunotherapy alone for resectable patients with clinical stage III/IV melanoma, with practice-changing clinical implications.^1,2^ However, investigation into the optimal agent and dosing schedule is ongoing.

## Present

We retrospectively analyzed PD-1 naive patients with resectable stage III/IV melanoma who received one dose of pembrolizumab (200 mg intravenous) prior to surgical resection. Of 51 patients, there were no grade 3/4 immune-related adverse events prior to surgery and no delays in surgery. There was prompt initiation of adjuvant therapy, along with appreciable rates of MPR (19.6%). For patients who achieved MPR, 5-year OS was 100%. In summary, in the largest case series of patients with oligometastatic melanoma treated with a single dose of neoadjuvant pembrolizumab, these data demonstrate that a shortened neoadjuvant dosing schedule may allow for an earlier pathological readout of responsiveness to anti-PD 1 monotherapy, while potentially avoiding some of the toxicity seen in both longer dosing schedules or with dual checkpoint blockade.^3^

## Future

A single agent/single dose anti-PD1 approach may be a potential treatment option in patients for whom neoadjuvant immunotherapy is indicated, particularly if there is concern regarding potential toxicity from doublet therapy and/or more frequent dosing. Additionally, the appreciable rates of MPR seen after single-dose pembrolizumab, and coinciding prolonged long-term survival, lend further credence to the response-driven approach, which has recently been studied in the NADINA and PRADO trials.^1,4^ While our current study reports a median follow-up time of 62 months, longer-term data from prospective neoadjuvant trials is necessary to give further insights. Additionally, future studies assessing more nuanced measures of treatment response beyond histopathologic characteristics, such as immune markers in the systemic circulation and sentinel node, may allow further tailoring of neoadjuvant treatment regimens. Expansion of this approach to earlier stage melanoma is also an active area of study. Preliminary data for single-dose neoadjuvant pembrolizumab in high-risk stage II melanoma from phase 2 clinical trial NCT03757689 was recently presented with promising results in patients with stage IIC disease; final clinical and translational data from this study will give further insights into efficacy and feasibility of the single-dose neoadjuvant immunotherapy approach.^5^

## References

[1] Blank CU, Lucas MW, Scolyer RA, et al. Neoadjuvant nivolumab and ipilimumab in resectable stage III melanoma. *N Engl J Med*. 2024;391(18):1696–708. 10.1056/NEJMoa2402604. 38828984 10.1056/NEJMoa2402604

[2] Patel SP, Othus M, Chen Y, et al. Neoadjuvant-adjuvant or adjuvant-only pembrolizumab in advanced melanoma. *N Engl J Med*. 2023;388(9):813–23. 10.1056/NEJMoa2211437. 36856617 10.1056/NEJMoa2211437 PMC10410527

[3] Shafique N, Farooq MS, Mattfeld V, et al. Single-dose neoadjuvant pembrolizumab in resectable metastatic melanoma. *Ann Surg Oncol*. 2025. 10.1245/s10434-025-18314-5. 40940600 10.1245/s10434-025-18314-5 PMC12534345

[4] Reijers ILM, Menzies AM, van Akkooi ACJ, et al. Personalized response-directed surgery and adjuvant therapy after neoadjuvant ipilimumab and nivolumab in high-risk stage III melanoma: the PRADO trial. *Nat Med*. 2022;28(6):1178–88. 10.1038/s41591-022-01851-x. 35661157 10.1038/s41591-022-01851-x

[5] Miura J, Farooq MS, Gimotty PA, et al. Neoadjuvant-adjuvant pembrolizumab in clinical stage IIB/C melanoma. *J Clin Oncol*. 2025;43(16_suppl):9502–9502. 10.1200/JCO.2025.43.16_suppl.9502.

